# Planning comparison of five automated treatment planning solutions for locally advanced head and neck cancer

**DOI:** 10.1186/s13014-018-1113-z

**Published:** 2018-09-10

**Authors:** J. Krayenbuehl, M. Zamburlini, S. Ghandour, M. Pachoud, S. Lang-Tanadini, J. Tol, M. Guckenberger, W. F. A. R. Verbakel

**Affiliations:** 1 Department of Radiation Oncology, University Hospital Zurich, Rämistrasse 100, CH-8091 Zurich, Switzerland; 2 Department of Radiation Oncology, Hôpital Riviera-Chablais, Avenue de la Prairie 3, CH-1800 Vevey, Switzerland; 3 Department of Radiotherapy, VU University Medical Center, De Boelelaan 1117, 1081 HV Amsterdam, The Netherlands

**Keywords:** Volumetric modulated arc therapy, Automated treatment planning, Head and neck carcinoma, Planning study, RapidPlan, Auto-planning, Raystation multicriteria optimization

## Abstract

**Background:**

Automated treatment planning and/or optimization systems (ATPS) are in the process of broad clinical implementation aiming at reducing inter-planner variability, reducing the planning time allocated for the optimization process and improving plan quality. Five different ATPS used clinically were evaluated for advanced head and neck cancer (HNC).

**Methods:**

Three radiation oncology departments compared 5 different ATPS: 1) Automatic Interactive Optimizer (AIO) in combination with RapidArc (in-house developed and Varian Medical Systems); 2) Auto-Planning (AP) (Philips Radiation Oncology Systems); 3) RapidPlan version 13.6 (RP1) with HNC model from University Hospital A (Varian Medical Systems, Palo Alto, USA); 4) RapidPlan version 13.7 (RP2) combined with scripting for automated setup of fields with HNC model from University Hospital B; 5) Raystation multicriteria optimization algorithm version 5 (RS) (Laboratories AB, Stockholm, Sweden). Eight randomly selected HNC cases from institution A and 8 from institution B were used. PTV coverage, mean and maximum dose to the organs at risk and effective planning time were compared. Ranking was done based on 3 Gy increments for the parallel organs.

**Results:**

All planning systems achieved the hard dose constraints for the PTVs and serial organs for all patients. Overall, AP achieved the best ranking for the parallel organs followed by RS, AIO, RP2 and RP1. The oral cavity mean dose was the lowest for RS (31.3 ± 17.6 Gy), followed by AP (33.8 ± 17.8 Gy), RP1 (34.1 ± 16.7 Gy), AIO (36.1 ± 16.8 Gy) and RP2 (36.3 ± 16.2 Gy). The submandibular glands mean dose was 33.6 ± 10.8 Gy (AP), 35.2 ± 8.4 Gy (AIO), 35.5 ± 9.3 Gy (RP2), 36.9 ± 7.6 Gy (RS) and 38.2 ± 7.0 Gy (RP1). The average effective planning working time was substantially different between the five ATPS (in minutes): < 2 ± 1 for AIO and RP2, 5 ± 1 for AP, 15 ± 2 for RP1 and 340 ± 48 for RS, respectively.

**Conclusions:**

All ATPS were able to achieve all planning DVH constraints and the effective working time was kept bellow 20 min for each ATPS except for RS. For the parallel organs, AP performed the best, although the differences were small.

## Background

In the past decade, intensity modulated radiotherapy (IMRT) and volumetric modulated radiotherapy (VMAT) became standard techniques for external beam radiotherapy treatments (EBRT) of many indications. The inverse optimization approach is an iterative process where optimization objectives are used in order to achieve the pre-defined clinical goals. Additionally, help structures are frequently defined to shape the dose distribution and further individualize and optimize the treatment plan. The complexity of the optimization increases with the number of organ at risks (OAR) and the number of target volumes. Head and neck carcinoma (HNC) is a typical complex case where a large number of OARs, typically 10–20, are surrounding the target volumes irradiated to different dose levels. This makes inverse planning optimization one of the most time consuming steps of the overall treatment planning process.

Additionally, plan quality may vary between planners and between clinical institutions. Plans produced by an experienced center may outperform those produced in a less experienced center [1] and the OAR sparing also depends on the planning target volume (PTV) dose homogeneity requirements [2]. Furthermore, evaluation of plan quality is often based on population-based dose volume histogram parameters (DVH), which neglect the nuances of an individual patients’ geometry and therefore do not achieve the optimal solution based on a patient-individual level [3]. In order to overcome these issues, optimization modules were developed in order to automate part or the entire optimization [4–9] process. They all aim at reducing the inter-planner variability, reducing the planning time allocated for the optimization process and finally improving the overall plan quality [10, 11]. Nowadays, automated treatment planning and/or optimization systems (ATPS) are in the process of broad clinical implementation. However, since ATPS have to be customized in order to fulfill the specific constraints required by different medical centers, it could be that an ATPS implemented at one institution will not necessary work for patients from another institution. The goal of this study was to compare different ATPS for HNC planning in a multicenter setting.

Additionally, it was evaluated if a model for automated planning developed by one institution could be used for planning cases of another institution using similar but not the same structures and planning goals. This multi-institutional planning comparison of five ATPS solutions is, to the best of our knowledge, the first of its kind.

## Methods

### Study design

In this multi-institutional planning study, five automated treatment planning systems used in 3 different institutes were evaluated:

1. Automatic Interactive Optimizer (AIO) (in-house developed) in combination with RapidArc version 13.7 from Eclipse (Varian Medical Systems, Palo Alto, USA) from hospital B [4, 12];
2. Auto-Planning version 14.0 (AP) from Pinnacle (Philips Radiation Oncology Systems) from hospital A [6];
3. RapidPlan version 13.6 (RP1) from Eclipse (Varian Medical Systems, Palo Alto, USA) using HNC model from hospital A;
4. RapidPlan version 13.7 (RP2) combined with scripting for automated setup of fields with HNC model from hospital B [13];
5. Raystation multicriteria optimization algorithm version 5 (RS) (RaySearch Laboratories AB, Stockholm, Sweden), from hospital C.

Ten randomly selected locally advanced HNC cases were chosen from each of two different institutes (A and B). Two cases from each group were used to familiarize with the target volume, concepts, dose constraints and to generate an automated planning strategy. A single optimization using the same strategy was performed for the remaining cases. Only these eight cases for each institution, overall 16 cases, were included in the planning comparison.

**Fig. 1:**
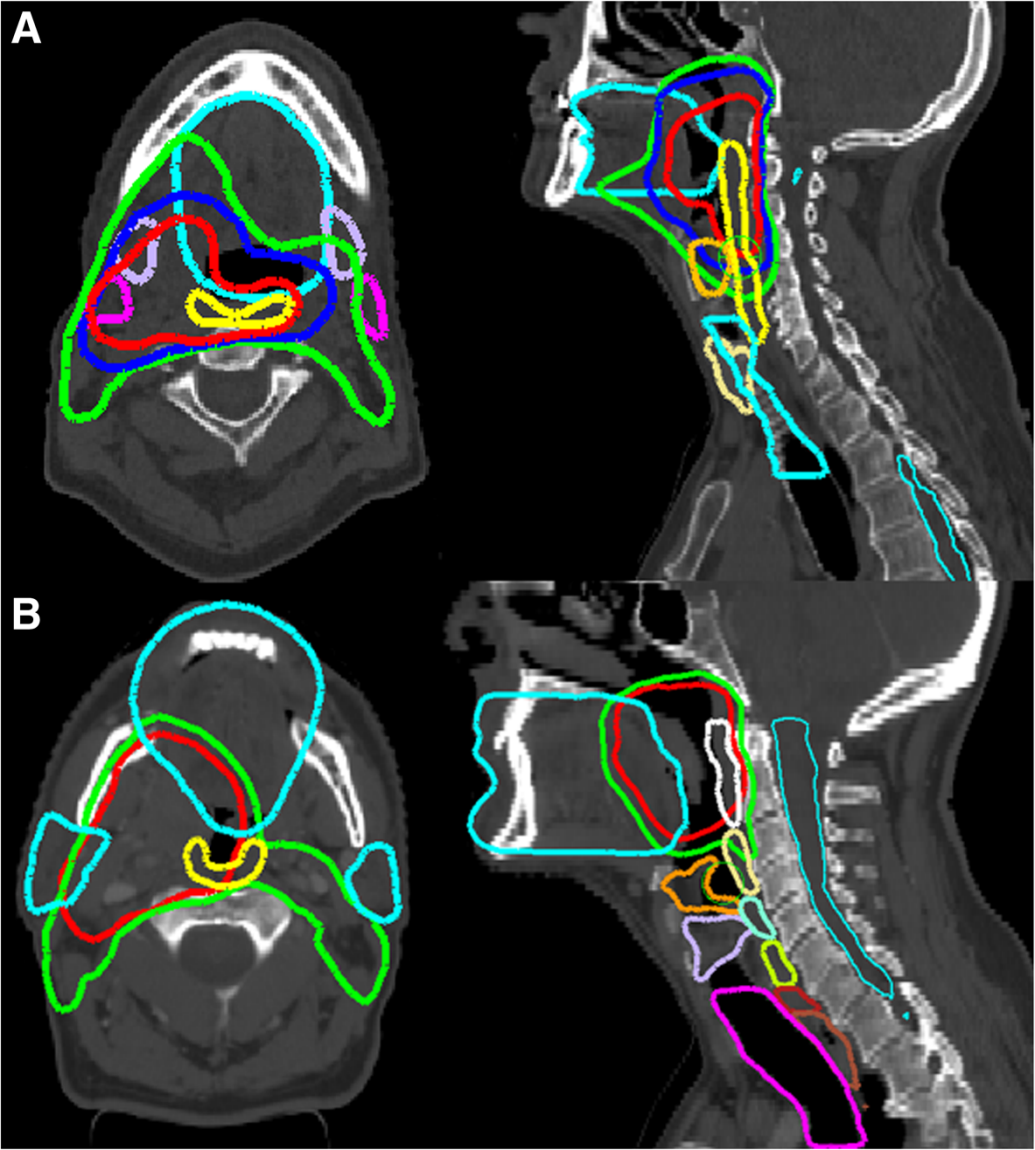
Example of one head and neck case from institution A (**a**) and one from institution B (**b**). In red, blue and light green the PTVs 70 Gy, 60 Gy and 54 Gy respectively as well as the OARs

Patients from institute A had three PTV dose levels, with doses of 70 Gy, 60 Gy and 54 Gy planned in 35 fractions using a simultaneous integrated boost (SIB), see Table 1 and Fig. 1. The dose was normalized to PTV 70Gy mean dose = 70 Gy.

**Table 1:** Targets, organs at risk and objectives used for plan optimization

Institution A	Institution B	
Prescription		
70 Gy, 60 Gy and 54 Gy	70 Gy and 54 Gy	
35 fractions with a simultaneous integrated boost		
Beam arrangement		
2 volumetric modulated arcs, 6MV photons		
PTVs		
PTV 70Gy Dmean = 70Gy	PTV 70Gy D95% = 98%	
PTV 70Gy V95% > 95%	PTV 70Gy V107% < 5%	
PTV 70Gy D2% > 75Gy	PTV 54Gy D95% > 98%	
PTV 60Gy V95% > 95%	PTV 54Gy V107% < 5%	
PTV 54Gy V95% > 95%		
Serial organs		
Brainstem Dmax < 54 Gy	Brainstem + 3 mm Dmax < 50 Gy	
Brachial Plexus D0.5cm^3^ < 60 Gy		
Mandible V70 Gy < 1 cm^3^	Mandible Dmax < 70 Gy	
Spinal cord < 45 Gy	Spinal cord + 3 mm Dmax < 54 Gy	
Parallel organs		
	UAT	Cricopharyngeal
Glottis		
		Larynx Lower
		Larynx Upper
Pharynx constrictor		
		Superior PCM
		Medial PCM
		Inferior PCM
Upper esophagus		Upper esophagus
		Upper Esophageal Sphincter
Thyroid		Thyroid
Trachea		Trachea
Oral cavity	Oral cavity	
Parotids	Parotids	
Submandibular glands	Submandibular glands	

*Abbreviation*: *PCM* pharyngeal constrictor muscles, *UAT* upper aerodigestve tract

Patients from institute B had two dose levels defined: 70 Gy and 54 Gy in 35 fractions using a SIB, see Table 1 and Fig. 1. The dose was normalized such that 95% of the PTV 70 Gy volume received 98% of the prescribed dose (70 Gy).

For both sets of patients, hard planning constraints were set for the PTVs and serial OAR, which had to be fulfilled by all planning system and patient cases, see Table 1. For the parallel OARs, the mean dose was asked to be kept as low as reasonably achievable. All plans were optimized with a 2 arc, 6 MV VMAT technique.

### Plan evaluation

Dose-volume histogram (DVH) parameters were calculated for the PTVs and each of the OARs listed in Table 1. Maximum doses for the serial OAR were reported but the differences were not considered in this planning comparison. This allows the optimization algorithm to further reduce dose to the parallel organs. The dose bath was evaluated based on the volumes covered by the 50 Gy, 30 Gy and 5 Gy isodose surfaces.

Doses to parallel OARs were evaluated based on their mean dose. A ranking for each parallel OAR was performed per patient. A rank of 1 was given for the ATPS achieving the lowest mean dose for a given OAR. Each ATPS achieving a dose within 3 Gy to the lowest achieved dose was given a ranking of 1, assuming that a dose difference < 3 Gy was not clinically relevant in our case [14–16]. The ATPS achieving a dose within 3 Gy – 6 Gy to the lowest achieved dose had a rank of 2, within 6 Gy – 9 Gy, a rank of 3, etc. The mean ranking was calculated for the individual swallowing muscles plus trachea and thyroid gland resulting in a structure called upper aerodigestive tract (UAT).

Finally, an overall mean ranking was taken by averaging the mean rank for parotid glands, submandibular glands and oral cavity.

The time required to generate a plan was evaluated and divided into 2 parts:

1. The effective working time was defined as the time required by the planner to generate a plan. This included the time needed for the definition of auxiliary structures and definition of bolus structures if a PTV reached the skin surface. For RS, it also included the user navigation through the Pareto-optimal plan database. This time didn’t include the time required for the optimization.
2. Optimization time, during which no interaction of the user was needed. For RS, it includes the automatic generation of Pareto-optimal plans and the final deliverable plan.

### Automated treatment planning system

#### AIO

Using the application programming interface (API) of the Eclipse treatment planning system, several scripts were developed to; create the PTV structures used in the optimization; generate ring structures around the PTVs; position two RapidArc fields and the isocenter positioned in the center-of-mass of the total PTV. The scripts automatically positioned fixed optimization objectives for the PTVs, serial OARs, and ring structures, while for each parallel OAR, 10 optimization objectives are positioned evenly spread among the volume axis. After this, the user has to manually open the optimization window of the treatment planning system to start the automatic interactive optimization (AIO) process. AIO is a program that automatically adapts the optimization objectives of the parallel OAR during the optimization process, keeping them at a fixed distance from the DVH-line at all times [12, 17].

#### Auto-planning

Auto-Planning (AP), included in Pinnacle 14.0 (Philips Radiation Oncology Systems), is a fully integrated module in the TPS, similar to the “manual” inverse optimizer module and has been previously described [6, 18]. Briefly, Pinnacle AP is a template-knowledge based treatment planning system. During AP, a plan is automatically loaded and the isocenter placed at the center-of-mass of the total PTV. The optimizer is than automatically run multiple times with the individual optimization goals, constraints and weights automatically added and adjusts the priority of clinical goals based on their probability of being achieved.

#### RapidPlan with a model from institute A (RP1)

Rapidplan (RP) is a knowledge-based automatic planning solution. A set of previously created plans representative of the internal hospital guidelines are fed into the program, which through a statistical modeling, creates a model. This model takes into account the geometrical and dosimetrical properties of the plans and is able to generate individualized constraints for the future plans taking into account their particular geometry, based on the library plans. Since RP is based on hospital-specific plans, the model is optimized to produce plans with a specific dose distribution and to accept OAR within a certain size and location. RP1 model was based on 83 clinically delivered HNC plans with SIB concept but variable dose levels created using 6MV photons and 2 full arcs. Since OAR definition from Institution A was different from Institution B, it was necessary to sum several OARs from Institution B before RP1 could be used.

#### RapidPlan with a model from institute B (RP2)

The same script as for AIO was used for the creation of the RapidPlan plans. However, instead of positioning all optimization objectives as a preparation for AIO, a script was developed to call a RapidPlan model from institute B to predict achievable OAR doses and position optimization objectives for the various targets and OARs. This model was based on 177 clinical patient plans, which is an extension of a previously made model [13].

#### Raystation

The MultiCriteria optimization algorithm MCO is a convex optimization problem [19] based on the approximation of the Pareto surface-based technique [20] where a set of Pareto-optimal plans is automatically generated and stored in a database for each patient. The user can navigate through this “Pareto-optimal” plans database and assess in real-time the tradeoff between different objective functions assigned to each anatomical structure. The desired plan that meets the clinical goals is then selected by the planner and generated to be delivered to the patient [21]. In this study, the geometry of planning targets volumes PTVs were replaced with convex approximation geometry in order to fully benefit from the MCO technique. The DVH-based functions were used as hard constraints in order to respect the clinical constraints.

### Statistical analysis

The mean dose to the parallel organs were reported as well as the standard deviation. The Wilcoxon signed rank test was used to compare the mean doses of the parallel organs of each planning system with those of the planning system that achieved the lowest mean dose. A *p* ≤ 0.05 was considered significant.

## Results

### Dose distribution

The detailed results for the five ATPS are included in Tables 2 and 3. Each planning system were able to be modified successfully to achieve the hard constraints for the PTVs and serial organs for each patients listed in Table 1. Small variations in serial OAR doses were observed between the different ATPS but were judged as clinically irrelevant.

**Table 2:** Detailed results for the PTVs, dose bath and serial organs

Structure			AIO		AP		RP1		RP2		RS	
			Mean	SD	Mean	SD	Mean	SD	Mean	SD	Mean	SD
Targets	PTV 70Gy	Dmean (Gy)	70.9	0.2	70.5	0.3	70.8	0.3	70.5	0.3	70.4	0.2
		V95% (%)	97.6	0.6	98.9	0.2	97.7	0.6	97.8	0.5	98.8	0.2
		D2% (Gy)	73.4	0.4	72.6	0.2	73.6	0.2	73.3	0.1	72.1	0.1
	PTV60Gy	V95% (%)	98.9	0.4	99.9	0.1	99.1	0.3	99.0	0.2	99.7	0.2
	PTV 54Gy	V95% (%)	98.2	0.4	98.7	0.6	98.3	0.4	98.8	0.3	98.0	0.6
Dose bath	Body	V50Gy (dm^3^)	1.02	0.34	1.12	0.34	0.96	0.30	1.00	0.32	0.94	0.32
		V30Gy (dm^3^)	2.50	0.62	2.32	0.66	2.12	0.56	2.38	0.61	1.88	0.43
		V5Gy (dm^3^)	6.39	1.28	7.12	1.40	6.83	1.35	6.54	1.40	6.64	1.36
Serial Organs	Brachial Plexus	D0.5 cm^3^ (Gy)	53.1	3.5	53.1	4.3	54.2	3.6	52.7	2.7	51.1	7.4
	Brainstem	Dmax (Gy)	30.2	15.5	27.4	13.6	26.2	14.7	30.6	12.8	23.7	13.4
	Mandible	Dmax (Gy)	67.8	4.6	66.6	5.5	68.0	7.9	67.1	12.0	67.0	3.2
	Spinal cord	Dmax (Gy)	40.7	0.8	41.5	2.2	41.3	2.1	40.8	1.3	36.0	2.9

*Abbreviations*: *AIO* automatic Interactive Optimizer, *AP* auto-planning, *RP* RapidPlan, *RS* Raystation, *V*_*95%*_ percentage volume receiving 95% of prescribed dose, *D*_*2%*_ dose corresponding to 2%, *D*_*0.5 cm*_^*3*^ dose corresponding to 0.5 cm^3^, *V*_*X Gy*_ volume receiving X Gy, *Dmax* maximal dose, *SD* standard deviation

**Table 3:** Detailed results for the parallel organs. Statistical significance was tested for each parallel organ group in comparison with the planning system that achieved lowest averaged mean dose (bold). Any 0.01 ≤ *p* ≤ 0.05 is indicated with a *, and *p* ≤ 0.01 is indicated with **

	AIO		AP		RP1		RP2		RS	
	Mean ± SD	Rank± SD	Mean ± SD	Rank ±SD	Mean ± SD	Rank ±SD	Mean ± SD	Rank ±SD	Mean ± SD	Rank ±SD
	(Gy)		(Gy)		(Gy)		(Gy)		(Gy)	
OralCavity	36.1 ± 16.8 **	2.56 ± 1.21	33.8 ± 17.8*	1.63 ± 0.62	34.1 ± 16.7**	1.81 ± 0.75	36.3 ± 16.2**	2.75 ± 1.34	**31.3 ± 17.6**	1.06 0.25
Parotid	21.9 ± 6.3	1.31 ± 0.6	**21.2 ± 5.9**	1.19 ± 0.40	21.6 ± 6.3	1.25 ± 0.45	22.8 ± 6.2	1.50 ± 0.73	21.4 ± 6.2	1.19 ± 0.54
Submand. Glands	35.2 ± 8.4	1.94 ± 1.34	**33.6 ± 10.8**	1.44 ± 0.81	38.2 ± 7.0 **	2.88 ± 1.41	35.5 ± 9.3	1.88 ± 1.09	36.9 ± 7.6*	2.38 ± 1.15
UAT	**38.8 ± 9.3**	1.49 ± 0.37	39.3 ± 9.4	1.86 ± 0.64	43.4 ± 7.6**	2.99 ± 1.01	39.7 ± 8.9	1.89 ± 0.7	40.4 ± 8.4	2.00 ± 0.70
Average		1.83 ± 1.06*		**1.53 ± 0.67**		2.23 ± 1.20**		2.00 ± 1.08*		1.66 ± 0.91

*Abbreviations*: *AIO* automatic interactive optimizer, *AP* auto-planning, *RP* RapidPlan, *RS* Raystation, *SD* standard deviation, *UAT* upper aerodigestve tract

The dose bath V_50 Gy_ and V_30 Gy_ was lowest for RS_,_ whereas AIO had the lowest V_5 Gy_, see Table 2. AP had on average the largest volume exposed to 50 Gy and 5 Gy. V_50 Gy_ and V_30 Gy_ were increased in comparison to RS by 1.9 ± 10.6% and 12.9 ± 18.0% (RP1), 6.1 ± 10.4% and 27.0 ± 13.7% (RP2), 8.7 ± 12.4% and 33.2 ± 13.0% (AIO), 18.5 ± 17.6% and 23.9 ± 17.8% (AP). V_5 Gy_ was increased in comparison to AIO by 2.4 ± 3.7% (RP2), 3.9 ± 4.7% (RS), 6.8 ± 9.9% (RP1) and 11.4 ± 12.8% (AP).

The mean dose results for the parallel organs are summarized in Table 3. Overall, AP had the best overall ranking for the parallel organs followed by RS (*p* = 0.20), AIO (*p* = 0.03), RP2 (*p* = 0.01), and RP1 (*p* < 0.01). When looking at each organ separately, the oral cavity mean dose was significantly lower with RS (31.3 ± 17.6 Gy) compared to AP (33.8 ± 17.8 Gy), RP1 (34.1 ± 16.7 Gy), AIO (36.1 ± 16.8 Gy) and RP2 (36.3 ± 16.2 Gy), *p* < 0.05. The lowest, respectively the highest, parotids mean dose was 21.2 ± 5.9 Gy (AP) and 22.8 ± 6.2 Gy (RP2) respectively. No significant differences were observed between the ATPS (*p* > 0.2). The submandibular glands mean dose was 33.6 ± 10.8 Gy (AP), 35.2 ± 8.4 Gy (AIO), 35.5 ± 9.3 Gy (RP2), 36.9 ± 7.6 Gy (RS) and 38.2 ± 7.0 Gy (RP1). Only RP1 and RS were significantly different to AP. In the subgroup of the UAT, large dose variations, up to 15 Gy, were observed for small structures such as the cricopharynx or the upper pharyngeal constrictor muscles between the ATPS. Averaging over all UAT structures reduced the differences. The lowest UAT mean dose was obtained with AIO (38.8 ± 9.3 Gy), AP (39.3 ± 9.4 Gy), RP2 (39.7 ± 8.9 Gy), RS (40.4 ± 8.4 Gy) and RP1 (43.4 ± 7.6 Gy). RP1 was the only ATPS having a significantly higher mean UAT dose compared to the ATPS achieving the lowest mean dose (AIO), *p* < 0.01.

### Planning time

The effective working time required after volume definition by the clinicians to the end of the optimization process was evaluated for every planning system. This time was kept below 2 min for each plan optimized with AIO and RP 2, see Table 4. The mean effective working time was increased by 3 ± 1 for AP, 13 ± 2 by RP1 and 116 ± 11 min by RS, respectively.

**Table 4:** Effective working time and optimization time

	AIO		AP		RP1		RP2		RS	
	Mean	StDev	Mean	StDev	Mean	StDev	Mean	StDev	Mean	StDev
Eff. working time (min)	< 2	< 1	5	1	15	2	< 1	< 1	116	11
Optimization time (min)	31	4	83	10	27	4	28	7	218	30

*Abbreviations*: *AIO* automatic interactive optimizer, *AP* auto-planning, *RP* RapidPlan, *RS* Raystation

## Discussion

This study presented a multi-institutional planning comparison study of five ATPS used in 3 different institutes, performed on 16 locally advanced head and neck cancer patients coming from two institutes. Although larger differences were observed for an individual patient, when looking at the mean results over all 16 patients, dosimetric differences between ATPS were generally small with Auto-Planning achieving the best ranking. Effective working time differed considerably more between ATPS, from 2 up till 116 minutes.

ATPS can be classified between automated optimization and automated planning, including optimization. The automated optimization can again be distinguished as optimization algorithm driven systems, such as AIO, AP and RS, were the objectives and/or priorities are automatically adjusted during the optimization and knowledge based planning systems based on plan libraries such as RP1 and RP2. The automated optimization algorithm driven systems can be easily modified to take into account possible changes in clinical protocols. This is not necessary the case for the knowledge based planning systems which rely on plans for a database of prior patients. Contrariwise, the use of a database allows a comparison between the predicted and achieved dose volume histogram [13].

The second classification can be performed based on automated planning where not only the optimization process is automated but also the field setup, gantry and collimator angles, the positioning of the isocenter and help structures such as bolus, rings structures or non-overlapping structures. This fully automated process was used by AIO, AP and RP2. RP1 and RS could also automate these planning process but it was not implemented.

After dose optimization, a single plan was generated by each ATPS except for RS where the user had to select a plan from a database of Pareto-optimal plans. This manual step will reduce the inter-planner standardization but will allow the user to choose the best dose trade-off between the targets and OARs.

Wu et al. [22] compared AP and RP, for oropharyngeal cancer patients and found that the plan quality from both systems was comparable. Differences between the two systems were in the range of 5%, which is in good agreement to the small differences observed in our study. To the best of our knowledge, no other ATPS comparisons are available.

The two sets of HNC were chosen to evaluate the flexibility of the different ATPS to take into account new structures, objectives and/or different dose levels. The model for AIO, AP and RS could be easily modified because they are based on a set of user pre-defined DVHs parameters, which were automatically adjusted during the optimization. However for the RS, the combination of objectives/constraints and the selection of their formulation had to be adjusted manually for each plan depending on the overlap between the PTV and OAR which affects directly the Pareto surface computation. This is not the case for RP models, which are based on previously generated site-specific plan libraries. In this case, the model had to be manually modified to take into account the structures not defined in the library. RP1 and RP2 were both used without considering whether a particular structure was an outlier. At later inspection, all OAR of the third case from group A were listed as “outside threshold values” for RP2 as the PTV_70Gy size of 585cm^3^ was above the 90 percentile value of the model. This could have a negative effect on the predictions. RP2 parotid gland doses were 7 Gy higher than for AP for this case. Similarly, when applying RP1 to the patients in the group B, the Glottis was marked as outlier for each single patient, and the swallowing muscles received with RP1 in these patients the highest mean dose. This was also the organ in which the highest differences between RP1 and the other ATPs were observed, clearly showing that the model was not able to predict the correct objectives for this case. This could be overcome by deciding that patients with such warning signs by RapidPlan should not be subject to automated planning. In spite of this, we compared how RP1 and RP2 performed for each organ with the data from the own institution and the external patient data. We did not notice large dosimetric differences, demonstrating that rapidplan also works for patients with slightly different structure sets and prescriptions.

The time required to generate VMAT or IMRT plans has been reduced in the past years by improvement of the available tools in planning system as well as automation of steps in the optimization process time. Nowadays, planning templates, scripts and optimization automation are available in TPS. This allows a gain of time on one side and a standardization of the plan quality at a high level on the other side. The effective working time for ATPS planned with VMAT was reported to be less than 10 min with iCycle [23] and less than 4 min with AP [6], but the overall time was not recorded. The effective working time reported are in the same order as those from our study. By adding scripting to the automated optimization processes, effective planning time could be reduced to less than 2 min with RP and AIO. Similar scripting tools are also available in AP but were not implemented. This might have led to a similar reduction of the effective working time.

RS required substantially more time to generate VMAT plans as the other ATPS mainly for two reasons. The first reason is the shape of the target that needs to be convex in order to allow the algorithm to efficiently converge on an optimal solution [17]. Earlier publications had shown that RS generated high quality plans in an efficient treatment planning time for convex target geometry [6, 18]. Therefore, each PTV geometry was approximated by a “more convex” or “less concave” geometry depending on the type of the nearest OAR (serial or parallel architecture). The second reason is that the HNC patients required a high-dimensional Pareto-surface approximation. Thus, the optimization time rises with the number of objective functions used during the optimization process. In our case, 20 objectives on average were used leading to a Pareto-surface approximation generated by 40 plans, as recommended by Craft et al. [20] for each patient. The optimization time was similar for RP for both institutions. AIO, which is running on the same system as RP, needed a few minutes longer to finish the optimization since the optimization is paused to automatically adjust the objectives. AP performs multitude steps of optimization and dose calculation where the objectives and help structures are automatically adjusted and created. This iterative process is time consuming and lasts typically between 1 h and 1.5 h. The optimization time is increased to three to 4 h with RS due to the reasons mentioned above. However, this approach allows the user to select the plan having the best balance between the targets and OARS dose. The optimization time mentioned above can be influenced by the number of users working in parallel on the server as well as its performances; therefore this parameter should be taken only as a rough estimation of the optimization time.

This study was focused on HNC treatment and whether similar results will be obtained for other sites still needs to be assessed.

## Conclusion

The results obtained for the five ATPS evaluated on two different set of HNC patients show that all ATPS were able to fulfill the hard constraints. For the parallel organs, AP achieved the best results followed by RS, AIO, RP2 and RP1. Nevertheless, the differences were small. The effective working time was reduced to less than 20′ for each ATPS, except RS, and could be reduced to less than 2′ when using scripting, which was the case for AIO and RP2.

## Abbreviations

AIO: Automatic interactive optimizer
AP: Auto-planning
ATPS: Automated treatment planning systems
D _0.5 cm_^3^: Dose corresponding to 0.5 cm^3^
D _2%_: Dose corresponding to 2%
Dmax: Maximal dose
DVH: Volume histogram parameters
EBRT: External beam radiotherapy treatments
HNC: Head and neck cancer
IMRT: Intensity modulated radiotherapy
OAR: Organ at risk
PCM: Pharyngeal constrictor muscles
PTV: Planning target volume
RP1: RapidPlan model from Hospital A
RP2: RapidPlan model from Hospital B
RS: Raystation
SD: Standard deviation
SIB: Simultaneous integrated boost
UAT: Upper aerodigestve tract
V _X Gy_: Volume receiving X Gy
VMAT: Volumetric modulated radiotherapy
