# Correction to: Planning comparison of five automated treatment planning solutions for locally advanced head and neck cancer

**DOI:** 10.1186/s13014-018-1176-x

**Published:** 2018-11-20

**Authors:** J. Krayenbuehl, M. Zamburlini, S. Ghandour, M. Pachoud, S. Tanadini-Lang, J. Tol, M. Guckenberger, W. F. A. R. Verbakel

**Affiliations:** 1 Department of Radiation Oncology, University Hospital Zurich, Rämistrasse 100, CH-8091 Zurich, Switzerland; 2 Department of Radiation Oncology, Hôpital Riviera-Chablais, Avenue de la Prairie 3, CH-1800 Vevey, Switzerland; 3 Department of Radiotherapy, VU University Medical Center, De Boelelaan 1117, 1081 HV Amsterdam, The Netherlands

## Correction

Following publication of the original article [1], the authors reported that one of the authors’ names was processed incorrectly. In this Correction the incorrect and correct author name are shown. The original publication of this article has been corrected.

Originally the author name was published as:

- S. Lang-Tanadini

The correct author name is:

- S. Tanadini-Lang

Furthermore, the authors reported that they were asked by RayStation to re-formulate statements concerning the multi-criteria optimization (MCO) algorithm in RayStation that could be misinterpreted. The original and revised statements are given below. The original publication of this article has been corrected.

Original statements:

Page 4:

The MultiCriteria optimization algorithm MCO is a convex optimization problem [19] based on the approximation of the Pareto surface-based technique [20] where a set of Pareto-optimal plans is automatically generated and stored in a database for each patient. The user can navigate through this “Pareto-optimal” plans database and assess in real-time the tradeoff between different objective functions assigned to each anatomical structure. The desired plan that meets the clinical goals is then selected by the planner and generated to be delivered to the patient [21]. In this study, the geometry of planning targets volumes PTVs were replaced with convex approximation geometry in order to fully benefit from the MCO technique. The DVH-based functions were used as hard constraints in order to respect the clinical constraints.

Page 7:

RS required substantially more time to generate VMAT plans as the other ATPS mainly for two reasons. The first reason is the shape of the target that needs to be convex in order to allow the algorithm to efficiently converge on an optimal solution [17]. Earlier publications had shown that RS generated high quality plans in an efficient treatment planning time for convex target geometry [6, 18]. Therefore, each PTV geometry was approximated by a “more convex” or “less concave” geometry depending on the type of the nearest OAR (serial or parallel architecture).

Revised statements:

Page 4:

The MultiCriteria optimization algorithm MCO is a convex optimization problem [19] based on the approximation of the Pareto surface-based technique [20] where a set of Pareto-optimal plans is automatically generated and stored in a database for each patient. The user can navigate through this “Pareto-optimal” plans database and assess in real-time the tradeoff between different objective functions assigned to each anatomical structure. The desired plan that meets the clinical goals is then selected by the planner and generated to be delivered to the patient [21]. In this study, the geometry of planning targets volumes PTVs were replaced with convex approximation geometry as a means to control the increased level of fluence modulation otherwise caused by a non-convex target shape. The DVH-based functions were used as hard constraints in order to respect the clinical constraints.

Page 7:

RS required substantially more time to generate VMAT plans as the other ATPS mainly for two reasons. The first reason is the technique employed in this study where each PTV geometry was approximated by a “more convex” or “less concave” geometry depending on the type of the nearest OAR (serial or parallel architecture). This additional planning step was introduced as earlier publications had shown that RS generated high quality plans in an efficient treatment planning time for convex target geometry [6, 17, 18].
